# Graphene Oxide Loaded with Protocatechuic Acid and Chlorogenic Acid Dual Drug Nanodelivery System for Human Hepatocellular Carcinoma Therapeutic Application

**DOI:** 10.3390/ijms22115786

**Published:** 2021-05-28

**Authors:** Kalaivani Buskaran, Mohd Zobir Hussein, Mohamad Aris Mohd Moklas, Mas Jaffri Masarudin, Sharida Fakurazi

**Affiliations:** 1 Laboratory for Vaccine and Immunotherapeutic, Institute of Biosciences, Universiti Putra Malaysia, Serdang, Selangor 43400, Malaysia; vaneey_88@yahoo.com; 2 Materials Synthesis and Characterization Laboratory, Institute of Advanced Technology, Universiti Putra Malaysia, Serdang, Selangor 43400, Malaysia; mzobir@upm.edu.my; 3 Department of Human Anatomy, Faculty of Medicine and Health Sciences, Universiti Putra Malaysia, Serdang, Selangor 43400, Malaysia; aris@upm.edu.my; 4 Department of Cell and Molecular Biology, School of Biotechnology, Universiti Putra Malaysia, Serdang, Selangor 43400, Malaysia; masjaffri@upm.edu.my

**Keywords:** graphene oxide, dual drug, nanodrug delivery, cancer therapy

## Abstract

Hepatocellular carcinoma or hepatoma is a primary malignant neoplasm that responsible for 75–90% of all liver cancer in humans. Nanotechnology introduced the dual drug nanodelivery method as one of the initiatives in nanomedicine for cancer therapy. Graphene oxide (GO) loaded with protocatechuic acid (PCA) and chlorogenic acid (CA) have shown some anticancer activities in both passive and active targeting. The physicochemical characterizations for nanocomposites were conducted. Cell cytotoxicity assay and lactate dehydrogenase were conducted to estimate cell cytotoxicity and the severity of cell damage. Next, nanocomposite intracellular drug uptake was analyzed using a transmission electron microscope. The accumulation and localization of fluorescent-labelled nanocomposite in the human hepatocellular carcinoma (HepG2) cells were analyzed using a fluorescent microscope. Subsequently, Annexin V- fluorescein isothiocyanate (FITC)/propidium iodide analysis showed that nanocomposites induced late apoptosis in HepG2 cells. Cell cycle arrest was ascertained at the G2/M phase. There was the depolarization of mitochondrial membrane potential and an upregulation of reactive oxygen species when HepG2 cells were induced by nanocomposites. In conclusion, HepG2 cells treated with a graphene oxide–polyethylene glycol (GOP)–PCA/CA–FA dual drug nanocomposite exhibited significant anticancer activities with less toxicity compared to pristine protocatechuic acid, chlorogenic acid and GOP–PCA/CA nanocomposite, may be due to the utilization of a folic acid-targeting nanodrug delivery system.

## 1. Introduction

Hepatocellular carcinoma or hepatoma is a primary malignant neoplasm that accounts for 75–90% of all liver cancer in humans [1]. Liver cancer has an assertive nature, which leads to poor survival rate and makes it a major public health problem in the world [2]. People from the Asia-Pacific region (East Asia and Southeast Asia) and Central and West African region are among those who are more prominently diagnosed with liver cancer [3]. The current treatments available for liver cancer are surgery, radiofrequency ablation, trans arterial chemoembolization and systemic chemotherapy [4]. There are side effects to these procedures where the systemic circulation of chemotherapy does not reside in the local accumulation of the diseased site but rather crosses cell barriers and damages the healthy cells as well [5]. Moreover, the procedure that is used needs an expert in the field to conduct the procedures which causes an increase in the treatment cost [6].

Nanotechnology was introduced as an alternative route to overcome the issues faced in conventional therapy. It creates new platforms that brings hope for next-generation cancer therapy. Nanotechnology has developed multiple therapies and diagnoses which enables early detection, diagnosis and effective delivery of anticancer drugs to avoid unwanted side effects and enhance the patient’s survival rate [7]. The dual drug nanodelivery method is also one of the initiatives in nanomedicine for cancer therapy. In dual drug nanodelivery researches, the co-delivery of two or more chemotherapeutic drug combinations via a nanocarriers delivery system was designed and formulated to enhance the antitumor activity against cancer cells [8].

Drug resistance is a foremost challenging factor in cancer therapy. The combination of various chemotherapeutic agents in one nanocarrier platform is an efficient method for cancer treatment [9]. Meanwhile, most of the anticancer drugs are low water soluble, hence it has low-bioavailability. So, the introduction of nanocarriers with combination drugs loading has the potential to maximize therapeutic effects, and minimize the toxic effects and drug resistance [10]. Chemotherapeutic drug delivery by a nanocarrier can induce the inhibition of cancer cells and increase the synergistic effect of multiple drugs as well as prevent drug resistance [11].

Graphene oxide (GO) is a derivative from graphene nanomaterial. The GO mainly consists of individual graphene sheets attached with oxygen functional groups on both the basal planes and edges [5]. It has a different structural model and contains various oxygen functional groups on its surfaces. The oxygen functional groups have been identified as hydroxyl and epoxy groups present on the basal plane whereas carboxyl, carbonyl, and phenol groups are at the edge of GO sheets. This unique two-dimensional structure of GO enables it to attract enormous attention for its relevance in anticancer drug delivery due to its high surface area and high loading capacity [5,7,12].

Phenolic compounds are a large group of secondary metabolites of plants. It is known for its properties that induce caspase-mediated apoptosis activity and enhance the cytotoxicity effect on various cancer cell lines [13]. These phenolic compounds are mainly responsible for apoptosis, the scavenging of radicals, antioxidant, and pro-oxidant characteristics of the antitumor activities. Protocatechuic acid (PCA) is a phenolic compound which is usually harvested from mainly green tea, *Olea europaea* (olives), *Hibiscus sabdariffa* (roselle), *Eucommia ulmoides*, *Citrus microcarpa Bunge* (calamondin), and *Vitis vinifera* (white wine grapes) [14,15].

Several studies have shown that PCA consists of major metabolites of complex polyphenols known as anthocyanins [16]. This has shown numerous physiological activities which are beneficial for health in reducing cardiovascular disease, anti-inflammatory properties, antioxidant, free radical scavenging activities, peroxidation inhibition and estrogenic/antiestrogenic activity [17]. Meanwhile, chlorogenic acid (CA) is usually found in coffee beans, teas, fruits and vegetables (apples, pears, carrots, tomatoes, and sweet potatoes). They are used for scavenging free radical activities, antioxidant, anti-inflammatory and anti-apoptotic activity in cancer cells [18].

Folate receptors are usually overexpressed in certain cancer cells due to the modification on the cell membrane [19]. This has turned out to be an advantage in nanomedicine when incorporating the folic acid (FA) into the nanoparticle formulation to target the drug delivered to the cancer cells. These folic acid ligands will ligate to the folate receptors on the cell membrane and enhance the drug nanocarrier endocytosis into the cells [20]. This active targeting method has been widely researched and utilized in the nanodrug delivery system.

Nanocomposites loaded with anticancer drugs can effectively prolong the drug circulation time in blood by channeling the drugs into the tumor site via the enhanced permeability and retention effect based on passive targeting [21]. Whereas, overexpression of receptor on the cancer cell membrane assists in ligand-based active targeting [22]. The multidrug delivery of passive and active targeting therapy has shown enhanced anticancer effects in the clinical application [23]. In this study, we have described the formation of a graphene oxide nanocomposite by the encapsulation of two drugs via nanoabsorption, incorporated with passive and active targeting for a nanodrug delivery system. Further, the characterization of the nanocomposites, in vitro cytotoxicity, cellular uptake, morphological changes, and finally, the anticancer toxicity and efficacy of passive and active targeting were investigated.

## 2. Results and Discussion

The nanoformulation of dual drug-loaded graphene oxide nanocomposites were successfully formulated in this study. Graphene oxide is a hydrophilic nanocarrier which possess characteristics such as a large surface area, biocompatibility, bioavailability, endocytosis ability, mechanical properties and low cytotoxicity [23]. The attachment of the functional groups on the basal and edge of the GO makes it a suitable nanocarrier for multiple drug attachment and modifications for the nanodrug delivery system. The aqueous solubility of the GO was enhanced by the PEGylation process [24]. The polyethylene glycol (PEG) conjugated GO will enhance the biocompatibility of the GO which enables the anticancer drug to bind to the graphene oxide surface through π–π stacking [25]. Despite this, it also increases the circulation half-life of the nanocarrier by escaping the reticuloendothelial system (RES) [26]. Both phenolic compounds of PCA and CA are known as an anticancer drugs [27]. The combination of the dual drug loading is speculated to give a better result in terms of killing the cancer cells. Besides, folic acid was attached to actively target the tumor site.

### 2.1. X-ray Diffraction Analysis of the Nanocomposites

**Figure 1:**
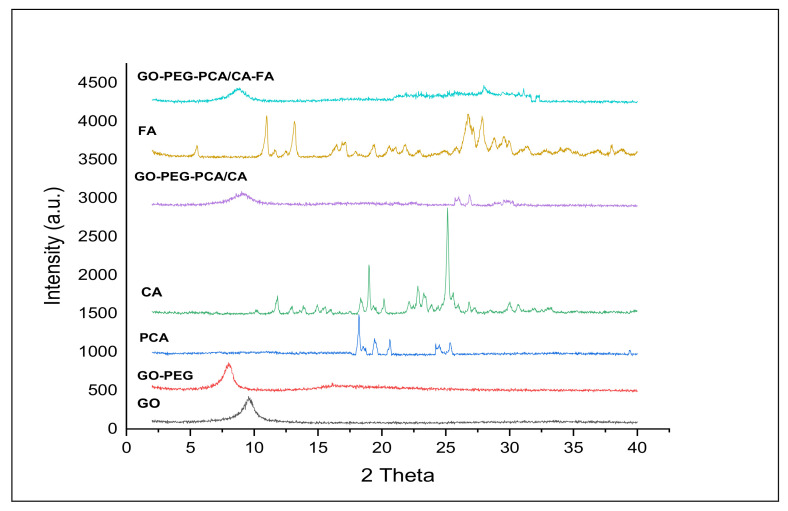
X-ray diffraction (XRD) patterns of graphene oxide (GO), graphene oxide-polyethylene glycol (GO-PEG), protocatechuic acid (PCA), chlorogenic acid (CA), graphene oxide coated PEG and loaded with protocatechuic acid+ chlorogenic acid (GOP-PCA/CA), folic acid (FA), and graphene oxide coated PEG and loaded with protocatechuic acid+ chlorogenic acid tagged with folic acid (GOP-PCA/CA-FA) nanocomposite.

Successful nanocomposites formulation was determined using X-ray diffraction analysis. In Figure 1, the reflections of graphene oxide (GO), graphene oxide conjugated PEG (GOP), protocatechuic acid (PCA), chlorogenic acid (CA), folic acid (FA) and nanocomposites (GOP-PCA/CA and GOP-PCA/CA-FA) are depicted. The PEG exhibited peaks at 19.21° and 23.26° and the GOP showed a highest spike for GO with a small hub between 2ϴ of 15°–25° because of the PEGylation process [28]. Both pristine PCA and CA drugs indicated XRD peaks to show some spikes in between 2ϴ of 15°–35°, which indicated the predicted organic component of the XRD patterns [8]. There are few reflections that can be observed which indicate the crystalline nature of the FA. Finally, GOP-PCA/CA and GOP-PCA/CA-FA nanocomposites show the spikes for GO presented to be broader and not as pointed as GOP, supported by the facts of the successful drug loading of PCA, CA and coating with FA.

### 2.2. Determination of Size Distribution and Zeta Potential Measurement of Nanocomposites

**Figure 2:**
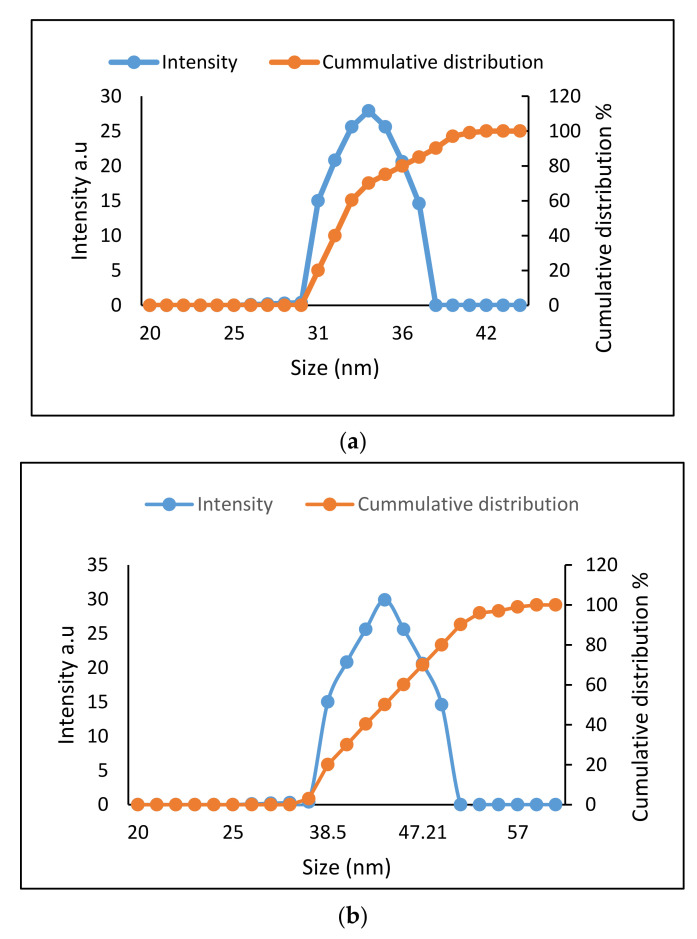
Particle size, relative and cumulative distributions of the (**a**) GOP-PCA/CA and (**b**) GOP-PCA/CA-FA nanocomposite.

The particle size and zeta potential of the nanocomposites were analyzed using dynamic light scattering (DLS) analysis. The particle size and zeta potential measurement of the nanocomposites were conducted in an aqueous solution. Figure 2a (GOP–PCA/CA) and Figure 2b (GOP–PCA/CA–FA) show the particle size distribution and cumulative distribution of nanocomposites. The hydrodynamic size of GOP-PCA/CA and GOP-PCA/CA-FA nanocomposites were found as peaks 34 ± 2.4 nm and 42 ± 3.9 nm, respectively, with an excellent polydispersity index (PDI ~0.28 and ~0.30). The FA coated to the GOP nanocomposite as a ligand resulted in the relatively larger size of the composite. Table 1 shows the zeta potential value of the nanocarriers and nanocomposites in different physiological systems. The size of the nanocomposites and surface charges determined the protein adsorption and cellular interaction with the physiological system [29]. The smaller size of nanocomposites enhances the bioavailability and aqueous stability, and at the same time escapes the filtration and clearance process by lymph systems as a foreign matter [30]. Besides, macrophages and phagocytes react strongly to the positively charged nanocomposite compared to those negatively charged [31]. In this case, the negatively charged nanocomposites have the potential to increase the circulation half-life of the material by evading the immune system.

### 2.3. Quantification of Dug Loading and Encapsulation Efficacy

Table 2 displays the percentage of PCA and CA drugs loading and encapsulation efficiency in the GOP–PCA/CA and GOP–PCA/CA–FA nanocomposites, respectively. The total dual drug loading was 42.37% in GOP-PCA/CA and 47.80% in GOP-PCA/CA-FA. The drug loading percentage observed in our study was found to be comparable with previously reported work utilizing GO as a nanocarrier. The loading ratio of the drug mainly depends on the distribution coefficient of the drug dissolved in solvent used and that adsorbed onto the surface of the graphene oxide nanocarrier [32].

### 2.4. Surface Properties Analysis

**Figure 3:**
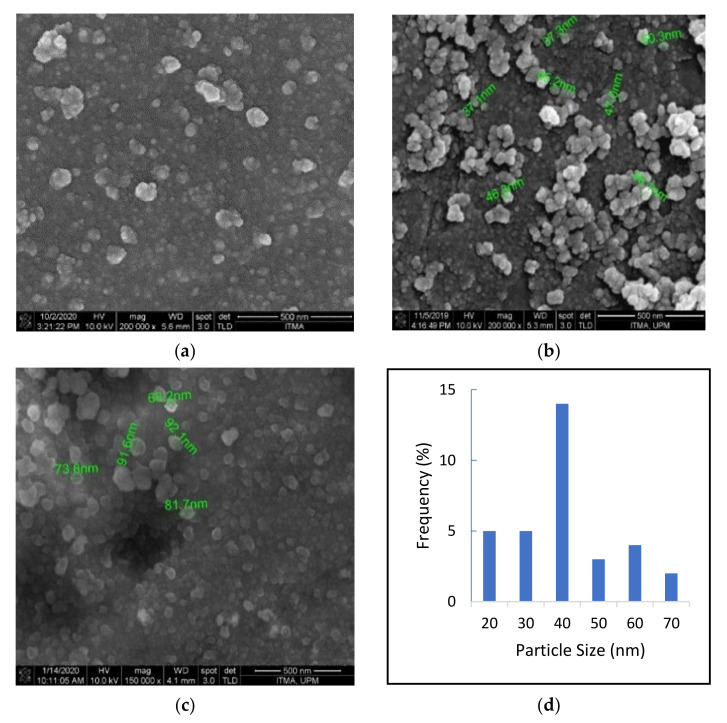

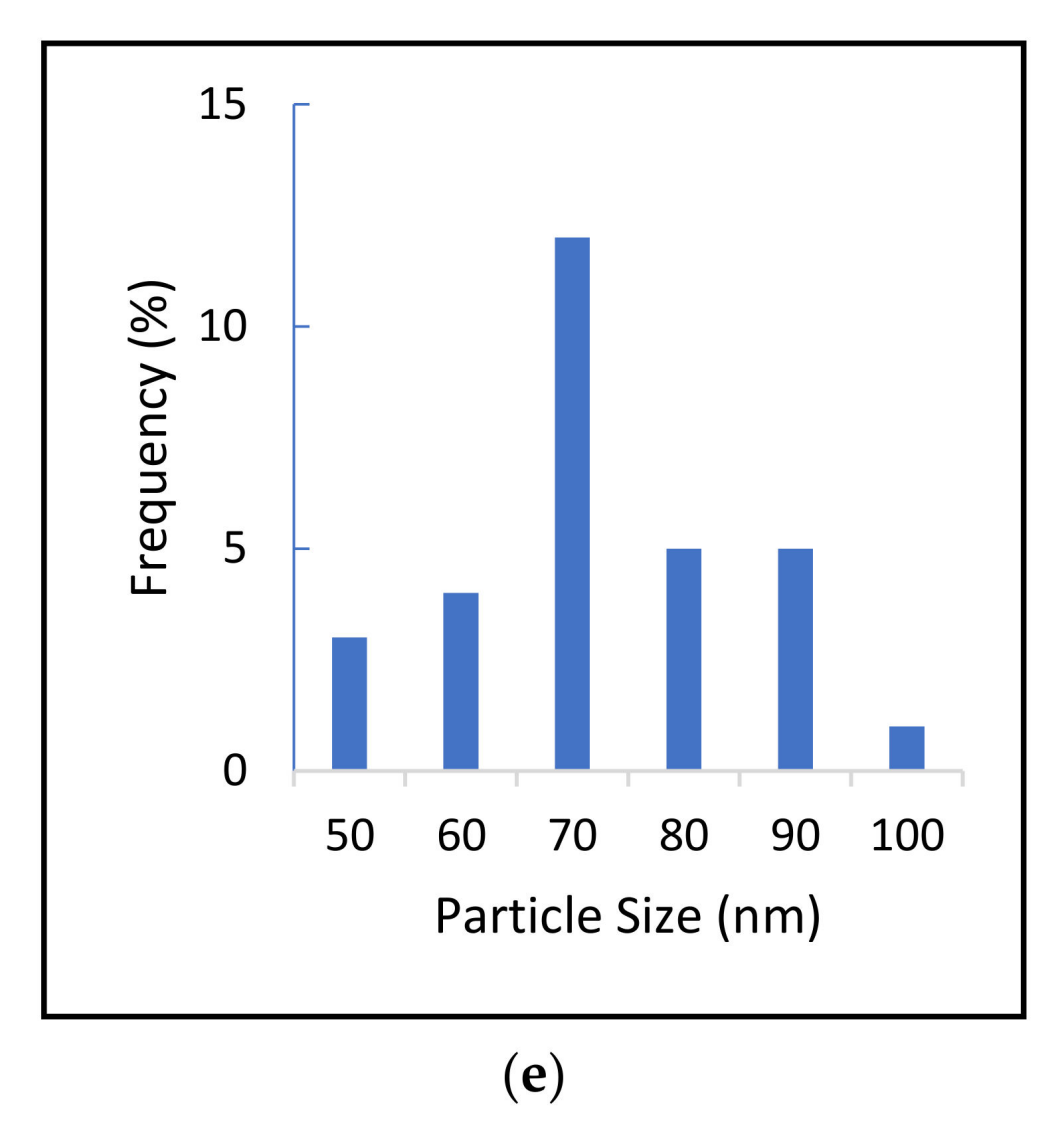
FESEM images of the (**a**) GOP, 200,000× (**b**) GOP-PCA/CA, 200,000× (**c**) GOP-PCA/CA-FA nanocomposite, 150,000× (**d**) GOP-PCA/CA particle size distribution graph (**e**) GOP-PCA/CA-FA particle size distribution graph.

The surface and morphology of the nanocomposites were observed using a field emission scanning electron microscope (FESEM). Figure 3a depicts the surface morphology of the GOP nanocarrier. Figure 3b shows interactions of the GOP-loaded pristine PCA and CA drugs (GOP-PCA/CA) entrapped on the graphene oxide. The sample displayed surface of self-aggregated structures with an average size around 30–50 nm, which is due to the nanocomposites aggregations. Figure 3c represents GOP-loaded PCA and CA drugs with folic acid-coated nanocomposite, (GOP-PCA/CA-FA) with an average size of 60–80 nm, which is relatively bigger due to the presence of FA as a coating agent. Figure 3d represents the particle size distribution of GOP-PCA/CA whereas Figure 3e represents the particle size distribution of GOP-PCA/CA-FA nanocomposite. Overall, the synthesized nanocomposites show a spherical shape, narrow size distribution and a smooth surface [23,33].

### 2.5. Protocatechuic Acid and Chlorogenic Acid Drug Release Study of Nanocomposites

**Figure 4:**
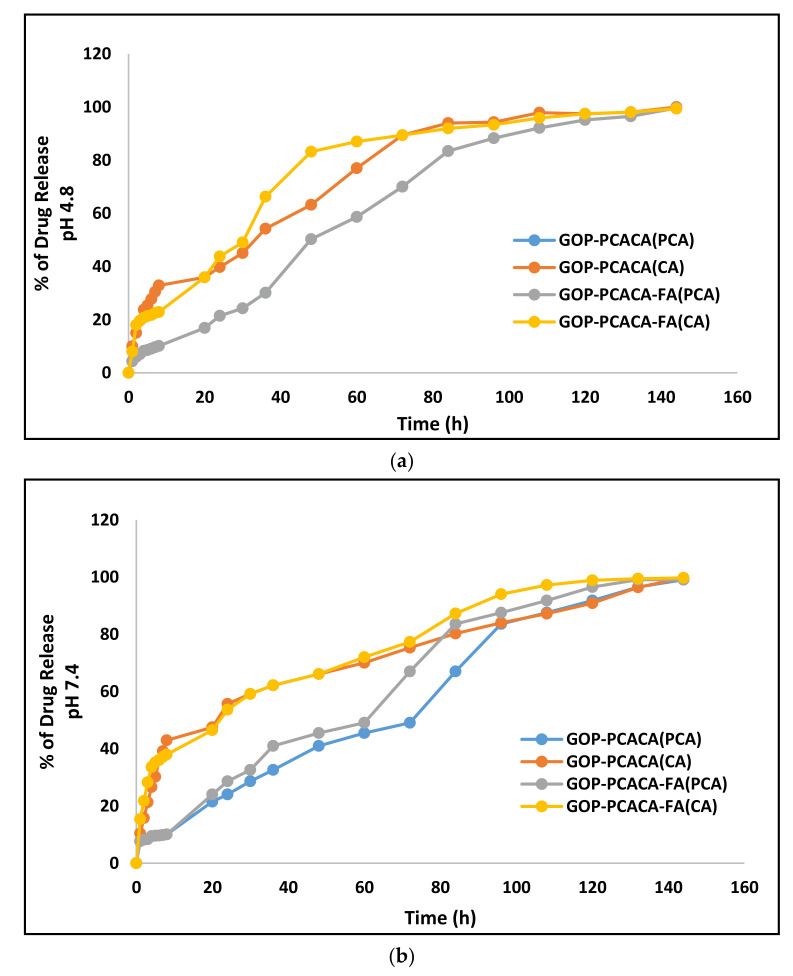
(**a**) In vitro release of PCA and CA from GOP-PCA/CA and GOP-PC/ACA-FA in phosphate buffer saline (PBS) solution in pH 4.8. (**b**) In vitro release of PCA and CA from GOP-PCA/CA and GOP-PCA/CA-FA in PBS solution in pH 7.4.

In vitro release studies of PCA and CA from GOP-PCA/CA and GOP-PCA/CA-FA nanocomposites were conducted in a human body replicated phosphate buffer saline (PBS) solution of pH 4.8 (intercellular lysosomal pH) and pH 7.4 (human blood pH) at 37 °C [23]. The absorption for PCA and CA were taken at 256 nm and 330 nm, respectively, as these are their individual lambda maxes [8]. Figure 4a represents the in vitro release of nanocomposites in pH 4.8 and Figure 4b shows the in vitro release of nanocomposites in pH 7.4. In both physiological conditions, PCA took about 120 h and CA took about 130 h for the complete release from the GOP-PCA/CA and GOP-PCA/CA-FA nanocomposites. Both drugs show the burst release of CA in pH 4.8 and pH 7.4 for the first 8 h. The maximum percentage of drug released by pH 4.8 was from CA, which in both nanocomposites were 89% at 72 h. Whereas at pH 7.4 the maximum percentage was also from CA, which was approximately 77% at 72 h. Interestingly, dual drug-loaded nanocomposites from GOP-PCA/CA and GOP-PCA/CA-FA show better drug release by chlorogenic acid than protocatechuic acid. The PCA drug release from GOP-PCA/CA(PCA) and GOP-PCA/CA-FA(PCA) are almost identitical. The release of PCA and CA at pH 4.8 may be due to the dissociation of two possible H-bonding interactions between the drug and the nanocarrier. These burst release phenomena are related to those drugs that were attached to the nanocomposite surface via the partial dissociation of hydrogen bonding [34]. Moreover, the arrangement of the drugs on the GOP nanocarriers and the size of the drugs influence the drug release. Cancer cells are generally associated with more acidic pH levels compared to the extracellular pH in normal tissues (7.4). This existing character in cancer cells may favor the drug delivery system. As it is, the sustained controlled drug release at pH 4.8 is considered to be the favorable condition for the anticancer treatment [35,36].

### 2.6. Cell Viability Effect of Nanocomposites on Normal and Cancer Cells

**Figure 5:**
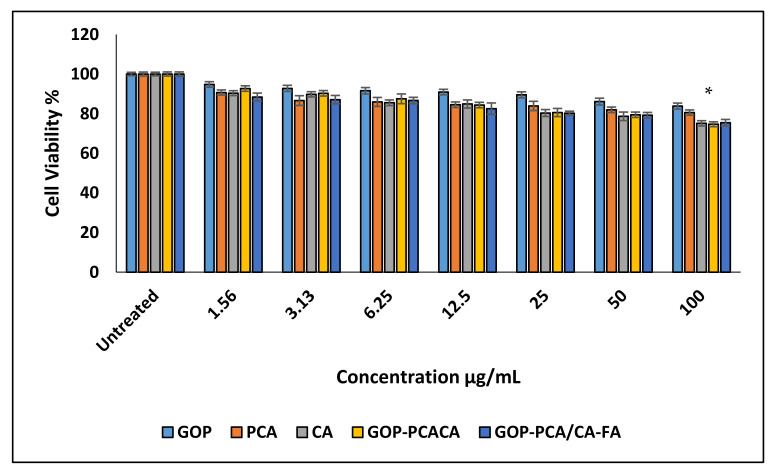
Cytotoxicity assay of graphene oxide-PEG (GOP), protocatechuic acid (PCA), chlorogenic acid (CA), GOP+ protocatechuic acid+ chlorogenic acid (GOP-PCA/CA), and GOP-PCA/CA coated folic acid (GOP-PCA/CA-FA) nanocomposite against normal HDFa dermal fibroblast cells at 72 h. Values are expressed as mean ± SD of triplicates. The significant difference (*p* < 0.05) * was determined between untreated HDFa cells and GOP, PCA, CA, GOP–PCA/CA and GOP–PCA/CA–FA nanocomposites by one-way ANOVA followed by Games–Howell post hoc tests.

**Figure 6:**
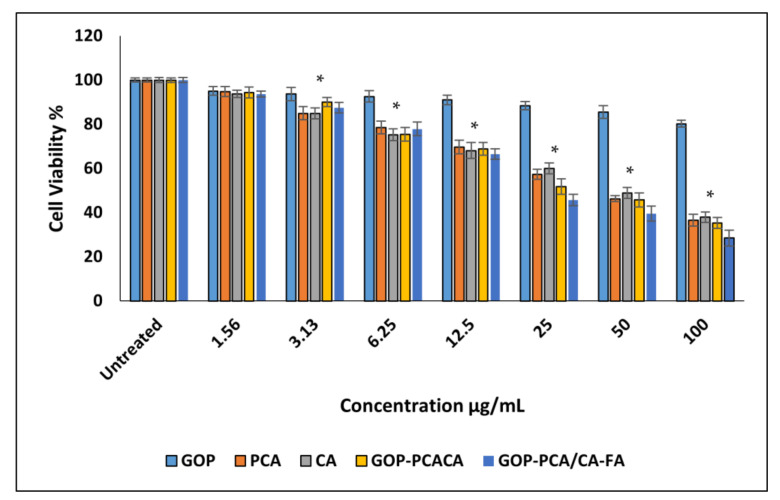
Cytotoxicity assay of graphene oxide-PEG (GOP), protocatechuic acid (PCA), chlorogenic acid (CA), GOP+ protocatechuic acid+ chlorogenic acid (GOP-PCA/CA), and GOP-PCA/CA coated folic acid (GOP-PCA/CA-FA) nanocomposite against HepG2 cells at 72 h. Values are expressed as mean ± SD of triplicates. The significant difference (*p* < 0.05) * was determined between untreated HepG2 cells and GOP, PCA, CA, GOP–PCA/CA and GOP–PCA/CA–FA nanocomposites by one-way ANOVA followed by Games–Howell post hoc tests.

Cytotoxicity screening was conducted, and toxicity was observed for up to 72 h on a normal human dermal fibroblast cell line HDFa (Figure 5) and human hepatocellular carcinoma HepG2 (Figure 6). It shows that cell viability at concentrations between 1.56 to 100 µg/mL exhibits no toxicity on all the cell lines with 80% of cell viability. The cytotoxicity screening was continued using the two different bioactive compounds used for the different nanocomposite formulations. Pure compounds of PCA and CA were used as a positive control and the negative control were cells incubated with media only. Cell viability of pristine PCA and CA and nanocomposites GOP-PCA/CA, and GOP-PCA/CA-FA screening against the HDFa cell shows as nontoxic to the normal cell.

This pattern changed once the pristine PCA and CA, GOP-PCA/CA and GOP-PCA/CA-FA nanocomposites were introduced to the HepG2 cell. The action of nanocomposites was observed to be dose-dependent on the HepG2 cell. The half-maximal inhibitory concentration (IC50) concentration was observed to be PCA at 40.78 ± 1.92 µg/mL and CA was 43.61 ± 1.74 µg/mL. However, in the dual drug delivery of GOP-PCA/CA, it is shown that toxicity is achieved at a concentration of 34.73 ± 1.04 µg/mL and followed by GOP-PCA/CA-FA at 26.79 ± 1.63 µg/mL. All concentrations are quantified in Table 3, which depicts the IC50 value for the nanocomposites on all the cell lines and the statistical analysis for the inhibitory concentration value. The dual drugs show better cytotoxicity compared to individual drugs.

Previous studies have been reported that PCA and CA drugs induce apoptosis and prevent the growth of tumor cells, with excellent anti-tumor, anti-carcinogenic, anti-HIV, anti-inflammatory and anti-bacterial properties [37,38]. Active targeting onto the cell surface receptor has been researched vigorously in cancer researches since many cancer cell types display the upregulation of tumor-specific receptors [39]. Targeting ligands specific for receptors cells is essential, considering their participation in cellular uptake mechanisms which may amplify the therapeutic response. One of the receptors that is overexpressed in multiple tumor cells is known to be folate receptor alpha subunit [40].

### 2.7. Plasma Membrane Integrity Analysis

**Figure 7:**
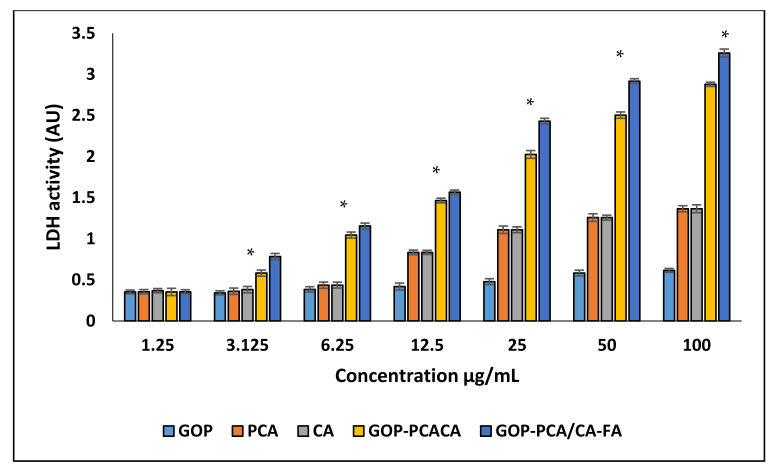
GOP nanocarrier, PCA and CA drugs, GOP–PCA/CA and GOP–PCA/CA–FA nanocomposites enhance lactate dehydrogenase release in HepG2 cells. All data are reported with mean ± SD of three independent experiments. The significant differences (*p* < 0.05) * were determined among GOP against PCA, CA, GOP–PCA/CA and GOP–PCA/CA–FA by one-way ANOVA followed by Games–Howell post hoc tests.

The plasma membrane integrity was determined by lactate dehydrogenase (LDH) being released into the extracellular culture medium by HepG2 cells. As shown in Figure 7, the experiment was conducted using a concentration range from 1.25–100 µg/mL on a HepG2 cell. The results proved that the influence of the cell membrane integrity of HepG2 cells responded in a dose-dependent manner. When cells were incubated with GOP, PCA, CA, GOP-PCA/CA and GOP-PCA/CA-FA at 12.5–100 µg/mL there was a significant increase in the level of LDH released, as compared to the control untreated cells. The endocytosis effect has been explained due to strong physical interactions of GO with the phospholipid layer, which causes loss of plasma membrane integrity [41,42]. An increase in LDH levels was also observed in our study, which was shown in a dose-dependent manner of graphene oxide (12.5 to 100 µg/mL).

### 2.8. Morphological Observation of Nanocomposite Cellular Uptake

**Figure 8:**
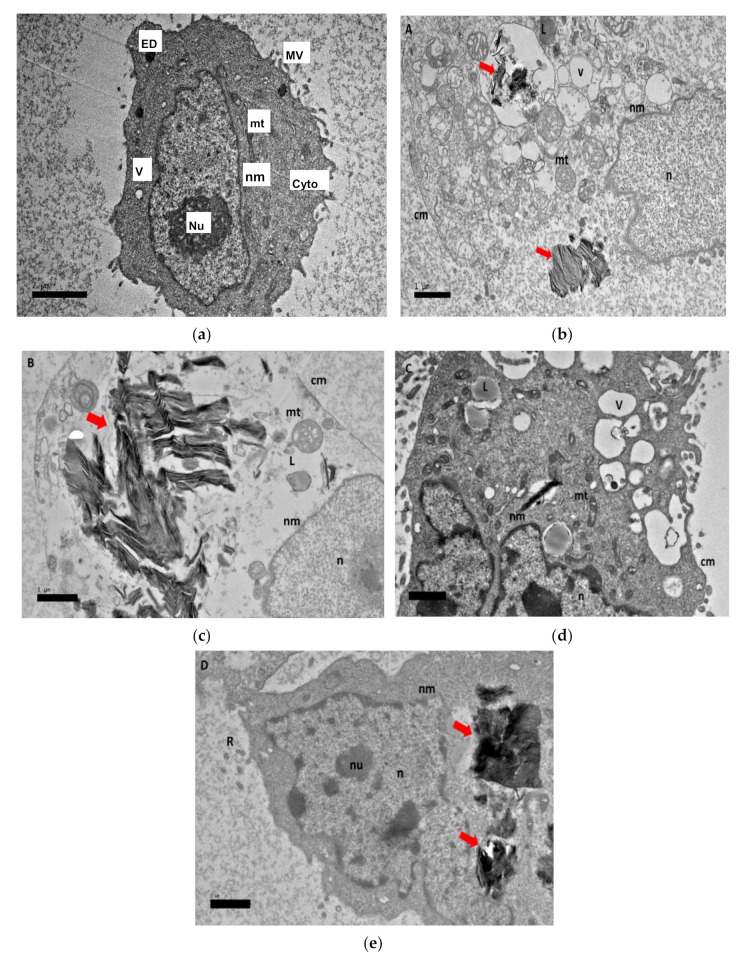
(**a**–**e**): Transmission electron micrographs of (**a**) untreated HepG2 cell as control for the nanocomposite cellular uptake by TEM at a magnification of 2000×. (**b**,**c**) GOP-PCA/CA, 1µ and (**d**,**e**) GOP-PCA/CA-FA, 1µ nanocomposite at 72 h. Cellular uptake of GOP-PCA/CA and GOP-PCA/CA-FA in HepG2 cell at 72 h by magnification of 3000×. Abbreviations: nu—nucleolus, n—nucleus, nm—nuclear membrane, mt—mitochondria, v—vacuole, cyto—cytoplasm and ED—electron dense. Scale bar 2.

Nanocomposites’ cellular uptake are depicted in Figure 8 using TEM observation. Figure 8a shows the untreated HepG2 cell. Figure 8b,c shows GOP-PCA/CA under 72 h of treatment, while Figure 8d,e are image of GOP-PCA/CA-FA after 72 h of treatment. These microgram images have confirmed the penetration of GO nanocomposites through the plasma membrane and its internalization into the cytoplasm, mitochondria and nucleus. It was reported that GO penetrated cells by piercing and mechanically disrupting the plasma membrane and aggregated inside the cells [42]. Cytoplasmic changes seen with TEM in Figure 8b–e were accredited to the characteristics associated with apoptosis. Once the nanocomposites enter the HepG2 cells at 72 h, the following changes could be observed in the cells, including the formation of numerous smaller vacuoles, vacuolization of mitochondria and loss of cristae, altered cytoplasm and lipid droplets accumulation. This morphological characterization of nanocomposites’ cellular uptake image offers an insight into apoptosis as the utmost cause of cell death [12].The apoptotic sequential occurrence such as chromatin condensation, blebbing and apoptotic body formation prior to the loss of membrane integrity can be observed in the cells [43].

### 2.9. In Vitro Localization and Accumulation of Nanocomposite

**Figure 9:**
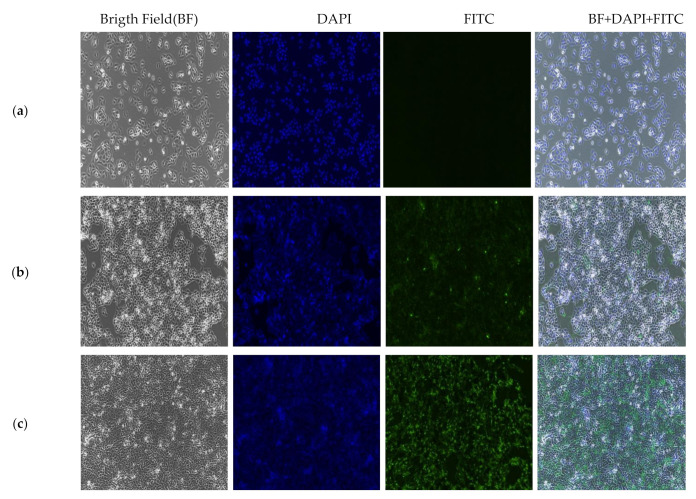
HepG2 cell treated with FITC-GOP-PCA/CA-FA conjugated at (**a**) 0 h, (**b**) 48 h and (**c**) 72 h under a florescent microscope. Magnification 100×.

The localization and accumulation of GOP-PCA/CA-FA conjugated fluorescein isothiocyanate (FITC) served as confirmation for real-time imaging to confirm the nanodrug is delivered at the targeted site. FITC is a rapid, simple, and sensitive fluorescein used to quantify cell-associated study by fluorometer [44]. As shown in Figure 9, FITC-GOP-PCA/CA-FA has confirmed that localization of the GOP-PCA/CA-FA occurred inside the HepG2 cells after 48 h and 72 h of incubation. This observation has suggested that GOP has the ability to deliver the PCA and CA drugs into HepG2 cells once they are intracellularly accumulated. FITC-GOP-PCA/CA-FA were effectively internalized, most likely through endocytosis. The green fluorescence was seen in the cytoplasm. Overlap of both signals of 4′,6-diamidino-2-phenylindole (DAPI) (blue) and FITC (green) confirms the localization occurs in the same cells [12,45].

### 2.10. Apoptosis Induced by Nanocomoposites in HepG2 Cells via Annexin V-FITC/Propidium Iodide (PI) Staining Analysis

The mode of cell death in HepG2 cells were analyzed using Annexin V/FITC and PI staining using flow cytometry [46]. In viable and healthy cells, Annexin V/FITC and PI stains do not bind the cells. However, when the cells undergo cell death induced by apoptosis, the following mechanism takes place: the externalization of the phosphatidylserine (PS) leaflet on the plasma membrane initiates the binding of the Annexin V/FITC alone which falls in the early apoptosis quadrant [47]. When the arrangement of the PS is flipped and the Annexin V/FITC binds the PS leaflet while PI binds the nucleus, this is categorized into the late apoptosis quadrant. Finally, in the necrosis quadrant PI stain, the nucleus and the cell membrane was ruptured [47,48].

**Figure 10:**
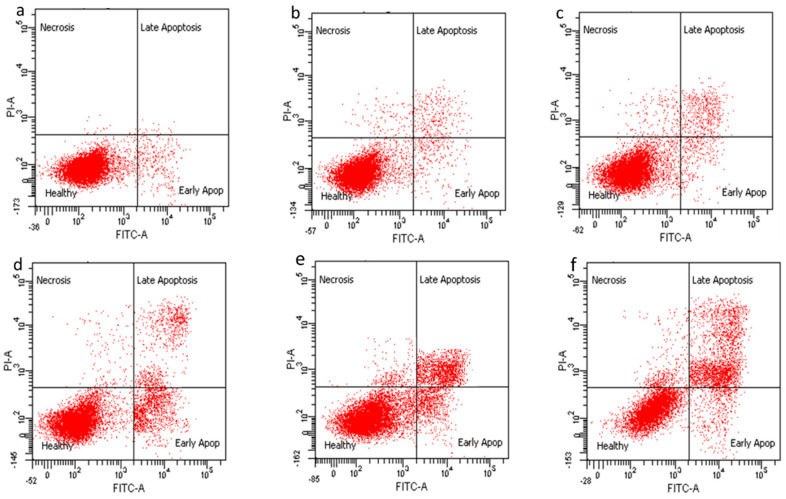
Dot plot of quadrant images of (**a**) untreated HepG2 cells, (**b**) GOP nanocarrier, (**c**) PCA, (**d**) CA, (**e**) GOP–PCA/CA nanocomposite and (**f**) GOP–PCA/CA–FA nanocomposite.

**Figure 11:**
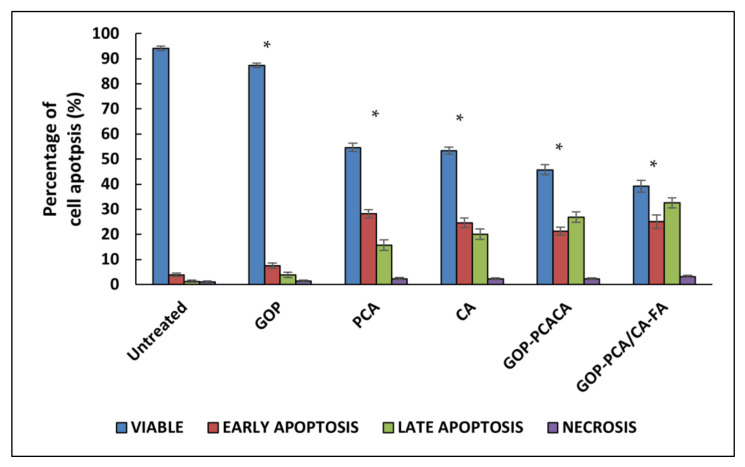
Histogram of the quantitative analysis of viable, early apoptosis, late apoptosis and necrosis HepG2 cells. Cells were treated with the GOP nanocarrier, PCA and CA drugs, GOP–PCA/CA and GOP–PCA/CA–FA nanocomposites at 40 µg/mL concentrations for 72 h. Values are expressed as mean ± SD of triplicates. The significant difference (*p* < 0.05) * was determined with untreated HepG2 cells against the GOP nanocarrier, PCA and CA drugs, GOP–PCA/CA and GOP–PCA/CA–FA nanocomposites by one-way ANOVA followed by Games–Howell post hoc tests.

Figure 10 shows a dot plot of quadrant images of the GOP nanocarrier, PCA and CA drugs, GOP–PCA/CA and GOP–PCA/CA–FA nanocomposites, whereas Figure 11 represents the histogram quantitative analysis of viable, early apoptosis, late apoptosis and necrotic percentage. The untreated HepG2 cells showed a percentage of 94.11 ± 0.38% viable cells, 3.28 ± 0.35% in early apoptosis, 1.27 ± 0.43% in late apoptosis and 1.1 ± 0.35% necrotic cells. Meanwhile, HepG2 cells incubated with the nanocarrier (GOP) showed the presence of 87.31 ± 0.36% viable cells, 7.55 ± 0.29% in early apoptosis, 3.83 ± 0.41% in late apoptosis and 1.39 ± 0.24% in necrotic stage.

The PCA drug show (54.61 ± 0.61% viable cells, 28.17 ± 0.42% in early apoptosis, 15.67 ± 0.38% in late apoptosis and 2.28 ± 0.11% necrotic cells) and CA (53.37 ± 0.66% viable cells, 24.62 ± 0.49% in early apoptosis, 20.11 ± 0.51% in late apoptosis and 2.19 ± 0.18% necrotic cells). Meanwhile, the GOP-PCA/CA nanocomposite (45.72 ± 0.29% viable cells, 21.33 ± 1.33% in early apoptosis, 26.79 ± 1.45% in late apoptosis and 2.23 ± 0.15% necrotic cells) GOP-PCA/CA-FA nanocomposite (39.14 ± 0.96% viable cells, 25.06 ± 1.89% in early apoptosis, 32.53 ± 1.76% in late apoptosis and 3.21 ± 0.47% necrotic cells). The comparison between the pristine drugs and nanocomposites shows significant *p* (˂0.05) reduction in GOP-PCA/CA-FA nanocomposite treated cells at the early apoptosis and late apoptosis stages.

### 2.11. The Effect of Nanocomposite on Cell Cycle Distribution in HepG2 Cells

**Figure 12:**
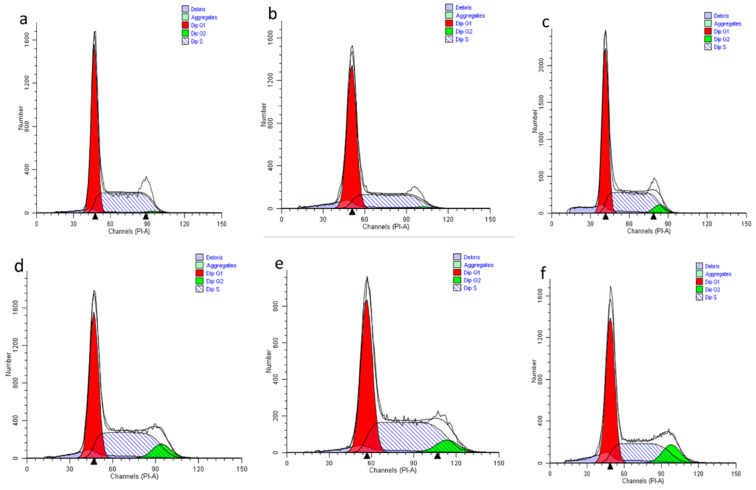
Representative cell cycle distribution of (**a**) untreated HepG2 cells, (**b**) GOP nanocarrier, (**c**) PCA, (**d**) CA, (**e**) GOP–PCA/CA nanocomposite and (**f**) GOP–PCA/CA–FA nanocomposite.

**Figure 13:**
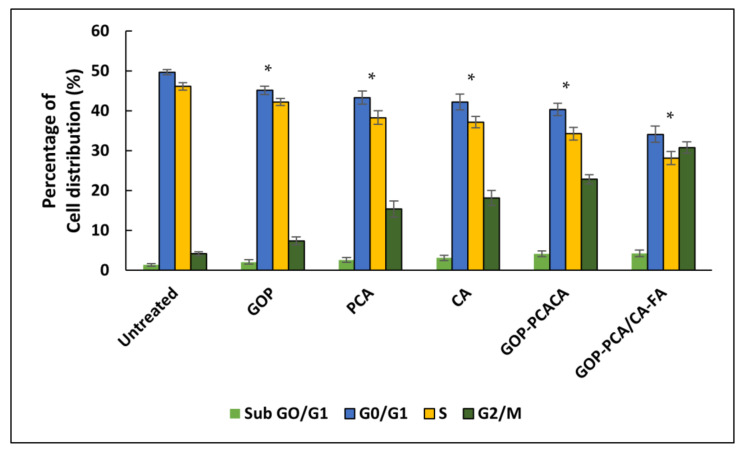
Histogram of the quantitative analysis of cell cycle arrest (%) in HepG2 cells. Cells were treated with the GOP nanocarrier, PCA and CA drugs, GOP–PCA/CA and GOP–PCA/CA–FA nano-composites at 40 μg/mL concentrations for 72 h. Values are expressed as mean ± SD of triplicates. The significant difference (*p* < 0.05) * was determined with untreated HepG2 cells against the GOP nanocarrier, PCA and CA drugs, GOP–PCA/CA and GOP–PCA/CA–FA nanocomposites by one-way ANOVA followed by Games–Howell post hoc tests.

The effect of pristine drugs (PCA and CA) and nanocomposites on cell cycle distributions were explored using PI staining by flow cytometry analysis [49]. The propidium iodide is known to stain the double stranded DNA. Figure 12 represents the cell cycle distribution and Figure 13 shows the percentages of the sub G0/G1, G0/G1, S and G2/M phases. The fluorescence intensity exhibited from flow cytometry was used to regulate the percentage frequency of cells at less than 2n as debris, sub G0/G1 as 2n, 2n–4n and 4n equally reflect the G0/G1, S and G2/M phases of the cell cycle [50].

The untreated HepG2 acts as a control exhibit regular DNA content of cell cycle distribution with G0/G1 at 49.72 ± 0.61% and the S phase at 46.12 ± 0.83%. The GOP nanocarrier exhibits almost similar G0/G1 and S phase distribution with 45.13 ± 1.07% and 42.23 ± 1.66%, respectively. However, when the HepG2 cells are treated with PCA, CA, GOP-PCA/CA and GOP-PCA/CA-FA nanocomposite, they show some significant changes in the cell cycle distribution.

The pristine drugs show the pattern of PCA (G0/G1 phase 43.28 ± 1.56%, S phase 38.29 ± 1.97% and G2/M phase 15.38 ± 1.66%) while CA (G0/G1 phase 42.19 ± 1.19%, S phase 37.17 ± 1.42% and G2/M phase 18.16 ± 1.63%). In the nanocomposites, significant reductions were observed in the G0/G1 phase (GOP-PCA/CA-40.34 ± 1.59%, GOP-PCA/CA-FA-34.12 ± 1.92%) and S phase (GOP-PCA/CA-34.27 ± 1.53%, GOP-PCA/CA-FA-28.19 ± 1. 86%). The accumulation of the G2/M phase was observed to be drastically increased in nanocomposite-treated cells which are 22.82 ± 2.01% and 30.78 ± 1.93%, respectively. While significant *p* (˂0.05) decreases were shown in the sub G0/G1 phase all the samples. These results suggested that HepG2 cells treated with nanocomposites were arrested at the G2/M phase, at parallel amplification in the accumulation at the sub G0/G1 phase and decrease in the G0/G1 and S phase of the cell cycle.

### 2.12. Nanocomposite Stimulates Mitochondrial Membrane Potential in HepG2 Cells

**Figure 14:**
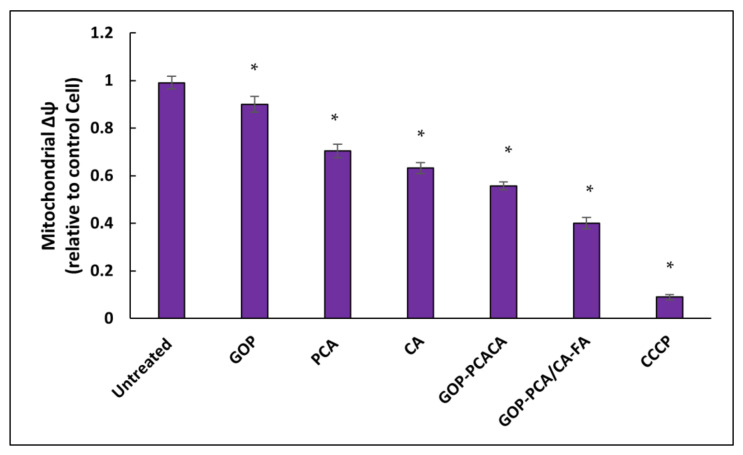
Mitochondrial membrane potential quantitative analysis for HepG2 cells. Cells were treated with the GOP nanocarrier, PCA and CA drugs, GOP–PCA/CA and GOP–PCA/CA–FA nanocomposites at 40 μg/mL concentrations and 10 µM of CCCP for 72 h. Values are expressed as mean ± SD of triplicates. The significant difference (*p* < 0.05) * was determined with untreated HepG2 cells against the GOP nanocarrier, PCA and CA drugs, GOP–PCA/CA and GOP–PCA/CA–FA nanocomposites by one-way ANOVA followed by Games–Howell post hoc tests.

The shift in mitochondrial membrane potential (Δψm) trigged by the drugs in the nanocomposites were regulated by a fluorescent probe (JC-1) [51]. Usually, the healthy mitochondria stained by JC-1 dye forms aggregates that exhibit red fluorescence, whereas the mitochondrial membrane, which tends to be permeable stained in JC-1 forms monomers that exhibit green fluorescence [52]. So, the ratio of red to green fluorescence shift in Δψm is displayed in Figure 14. The untreated HepG2 cells serve as a control composed of 0.99 ± 0.031 ratio which translates as high membrane potential. Next, the GOP nanocarrier showed a significant *p* (˂0.05) decrease in 0.90 ± 0.027 ratio compared to untreated cells.

When the pristine drugs (PCA and CA) and nanocomposites (GOP-PCA/CA and GOP-PCA/CA-FA) where stained with JC-1, there were remarkable shifts in the ratio of the red fluorescence to the amplification of green fluorescence. When HepG2 cells were treated with pristine PCA and CA, the mitochondrial membrane potential decreased by 0.70 ± 0.021 and 0.63 ± 0.023. This similar pattern observed in the cells treated with GOP-PCA/CA and GOP-PCA/CA-FA nanocomposite showed a decrease in 0.56 ± 0.031 and 0.40 ± 0.019 ratio, respectively. The carbonyl cyanide m-chlorophenylhydrazone(CCCP) serve as a positive control; they generated a non-apoptotic red fluorescence to green fluorescence ratio with 0.09 ± 0.016. The significant differences *p* (˂0.05) were compared between the untreated cells, and nanocomposites exhibited a significant decrease in the ratio of mitochondrial membrane potential.

### 2.13. Nanocomposite Induces Intracellular Reactive Oxygen Species Generation in HepG2 Cells

**Figure 15:**
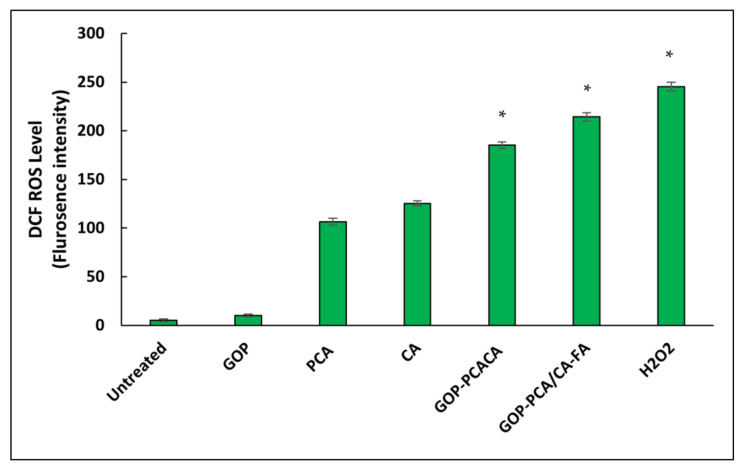
Reactive oxygen species (ROS) production in HepG2 cells were treated with the GOP nanocarrier, PCA and CA drugs, GOP–PCA/CA and GOP–PCA/CA–FA nanocomposites and H_2_O_2_ at 40 μg/mL concentrations for 72 h. Values are expressed as mean ± SD of triplicates. The significant difference (*p* < 0.05) * was determined with untreated HepG2 cells against the GOP nanocarrier, PCA and CA drugs, GOP–PCA/CA and GOP–PCA/CA–FA nanocomposites by one-way ANOVA followed by Games–Howell post hoc tests.

Intracellular reactive oxygen species (ROS) is important in facilitating cytotoxicity induced by chemotherapeutic drugs [53,54,55]. Thus, Figure 15 displays the effect of drugs and nanocomposites on ROS generation in HepG2 cells after 72 h of treatment using dichloro-dihydro-fluorescein diacetate (DCFH-DA) flurogenic dye. The untreated HepG2 cells serve as a negative control while exposing HepG2 cells to H_2_O_2_ serves as a positive control. The treatment of HepG2 treated with GOP induces a significant increase in the level of ROS productions compared to untreated cells. A gradual increase in ROS generation was observed when cells were treated with PCA and CA drugs; the GOP-PCA/CA and GOP-PCA/CA-FA nanocomposite, respectively. Interestingly, in GOP-PCA/CA and GOP-PCA/CA-FA, the intracellular ROS generation in HepG2 was increased from 185.17 ± 4.18% to 214.27 ± 2.98%. The results show that there are significant changes in ROS generation between the untreated and treated HepG2 cell. In the comparison between untreated cells and the treated nanocarrier, drugs and nanocomposites exhibit a significant increase in the value of ROS generation, respectively.

The nanocomposites of combination drugs show a remarkable result in treating HepG2 cells compared to individual drugs. The active targeting in nanocomposites was favored in achieving a better cytotoxicity value at a lower concentration in HepG2 cells. The folic acid-coated nanomaterial showed significant cellular uptake in the cancer cell. The nanocomposite not only forces HepG2 cells to undergo apoptosis, but triggers interconnected networks in the HepG2 cell death mechanisms. HepG2 cells undergo cell cycle mechanisms to be arrested at the G2/M phase when incubated with nanocomposites. These nanocomposites are capable of depolarizing the mitochondrial membrane potential and triggering apoptosis through ROS generation.

## 3. Materials and Methods

### 3.1. Materials

Graphite powder, sulphuric acid (H_2_SO_4_ 98%), phosphoric acid (H_3_PO_4_), potassium permanganate (KMnO_4_), PEG4000, N-(3-Dimethylaminopropyl-N′-ethylcarbodiimide hydrochloride (EDC), 240 mg N-hydroxysulfosuccinimide (NHS), hydrogen peroxide, phosphate buffer solution, methanol, and ethanol were all sourced from Sigma Aldrich, St. Louis, MO, USA. Pristine protocatechuic acid (PA) and chlorogenic acid (CA) was purchased from Sigma Aldrich, USA. LDH test-kit (CytoTox 96^®^ Non-Radioactive Cytotoxicity Assay, Promega Co., Madison, WI, USA), Annexin V/FITC Apoptosis Detection Kit and CycleTEST TM PLUS DNA Reagent Kit from (BD Biosciences, San Jose, CA, USA), JC-1 Mitochondrial Membrane Potential Assay Kit (Abnova, Middlesex County, NJ, USA), OxiSelect™ Intracellular ROS Assay Kit (Green Fluorescence) (Cell BioLabs, Inc., San Diego, CA, USA).

### 3.2. Cell Lines

Human dermal fibroblast cells (HDFa) and human hepatocellular carcinoma cells (HepG2) have been sourced out from ATCC (Manassas, VA, USA). The HDFa dermal fibroblast was grown in a fibroblast basal medium with a Fibroblast Growth Kit–Serum-Free (ATCC^®^ PCS-201-040). HepG2 cells were grown in Eagle’s Minimum Essential Medium with 10% *v*:*v* fetal bovine serum (FBS), 1% *v*:*v* penicillin (100 units/mL) and streptomycin (100 μg/mL) (Nacalai Tesque, Kyoto, Japan). The cells were cultured at 37 °C in a humidified 5% CO_2_ incubator. After 24 h when the cells reached 80–90% confluency, they were trypsinized using 0.25% trypsin/1 mM-Ethylenediaminetetraacetic acid (EDTA) (Nacalai Tesque, Kyoto, Japan) for further experiments.

### 3.3. Synthesis of Graphene Oxide and Conjugation with Polyethylene Glycol

Graphene oxide was prepared according to the modified Hummer method. Graphene oxide was prepared using 3 g of graphite power was mixed with 400 mL of concentrated acids (sulfuric acid, (H_2_SO_4_, 360 mL) and phosphoric acid (H_3_PO_4_, 40 mL)). The mixture was then added with 18 g of potassium permanganate (KMnO_4_). Approximately 700 mL of deionized water was added to the solution and stirred at 50 °C for 12 h. The temperature was reduced to 35 °C with ice cubes once the suspension was added with 3 mL of hydrogen peroxide. The mixture was washed to eliminate the excess materials by filtration process. The remaining solid was washed with 200 mL of HCl three times. The solution was then centrifuged and was washed with deionized water three times to purify the solid mixture. The sample was sonicated for 30 min. Neutralized graphene oxide was dried and later dialyzed with a dialysis bag (7000 Da). The GO conjugated the PEG by an esterification reaction between the carboxylic acid group and hydroxyl group. The GO suspension (2 mg/mL) was mixed in 20 mL of sodium hydroxide. The sample was centrifuged to produce GO carboxylic acid (GO-COOH). The activated carboxylic acid group in GO was catalyzed using 400 mg of N-(3-Dimethylaminopropyl-N’-ethylcarbodiimide hydrochloride (EDC) and 240 mg of N-hydroxysulfosuccinimide (NHS) and stirred for 24 h. This was followed by 1.5 g of PEG4000, which was added to the above suspension and constantly stirred overnight. Finally, the suspension was washed with deionized water and centrifuged to obtain GO conjugated PEG (GOP) [23].

### 3.4. Protocatechuic Acid and Chlorogenic Acid Loading on Graphene Oxide with PEG and Coated with Folic Acid

Protocatechuic acid (PCA) and chlorogenic acid CA (2.5 g in each) was loaded into 100 mL of GOP nanocarrier solution. The samples were stirred for 24 h followed by being thoroughly washed with deionized water and dried at 40 °C. The flaky material was then grounded into powder and resuspended in 50 mL of 1% folic acid solution and stirred for 24 h. Later, the sample was centrifuged at 8000 rpm, 25 °C for 15 min. The samples were then washed thoroughly with deionized water and dried in the oven at 40 °C. The resultant materials were then subjected to physiochemical characterization of GOP-PCA/CA and GOP-PCA/CA-FA.

### 3.5. Synthesis of FITC-Labelled GOP-PCA/CA-FA

The FITC-labelled GOP-PCA/CA-FA nanocomposite was prepared by dissolving 1 mg of FITC powder into 1 mL of DMSO as a stock solution and out of that, 50 µL of the FITC solution was then added into 200 µL of the GOP-PCA/CA-FA nanocomposite and incubated for 20 min at 24 °C. The process must be conducted away from light since FITC is light sensitive. The mixture was thoroughly mixed to ensure a homogeneous suspension was obtained. The FITC-GOP-PCA/CA-FA nanocomposite solution was covered with aluminum foil to protect it from exposure of light and stored at 4 °C for further analyses.

### 3.6. Physicochemical Characterization of Nanocomposites

The characterizations of the GOP-PCA/CA and GOP-PCA/CA-FA nanocomposites were conducted in dynamic light scattering (Nanosizer, Malvern, NanoS, Worcestershire, UK) for particle size distribution, polydispersity index (PDI) and zeta potential; the X-ray Diffraction X 6000 (Shimadzu, Tokyo, Japan) for crystalline phase analysis used a 2°–60° range using CuKα radiation (λ = 1.54060 Å) at 40 kV and 30 mA. Field emission scanning electron microscope (FESEM) images of the samples were recorded using a field emission scanning electron microscope, NOVA NANOSEM 230 model, (Hillsboro, OR, USA).

### 3.7. Encapsulation Efficacy and Drug Loading Content Analysis

The drug encapsulation efficacy and loading content of PCA and CA percentage in the nanocomposite were quantified using a Waters HPLC model 2695 equipped with an Agilent C18 column, photodiode array (PDA) detector and Empower software (Waters, Milford, MA, USA). For the stock solution of nanocomposites, they were prepared in methanol with a concentration of 0.5 mg/mL. Working standard solutions for nanocomposites were prepared within ranging concentrations of (10–300 µg/mL in acetyl nitrate) by diluting the stock solution in pure methanol. The samples around 20 µL were injected into the column and detected at 210 nm. The encapsulation efficiency (%EE) and the loading content (%LC) of GOP-PCA/CA and GOP-PCA/CA-FA nanocomposites were calculated using the following formula:

EE (%) = (Total nanocomposites-free drugs)/(Total nanocomposites with drugs) × 100(1)

LC (%) = [The weight of drug in nanocomposites/the weight of nanocomposites] × 100(2)

### 3.8. In Vitro Drug Release of Protocatechuic Acid and Chlorogenic Acid from Nanocomposites

The drug release from nanocomposites were conducted in a Perkin Elmer UV/Vis spectrophotometer (Model Lambda 35, Mundelein, IL, USA). The lambda max for PCA (256 nm) and CA (330 nm) wavelength were used to determine the in vitro drug release kinetic studies. For the in vitro release studies, approximately 5 mg of each PCA and CA drug was added into 5 mL of pH 7.4 (blood) and pH 4.8 (intracellular lysosomal pH) buffers separately and the release profile was determined by taking absorption at different time intervals from 0 h until 144 h (6 days).

### 3.9. In Vitro Cell Cytotoxicity Assay

The cell cytotoxicity evaluation of nanocomposite treatments was conducted using a methylthiazol tetrazolium (MTT) assay. The normal human dermal fibroblast (HDFa) and human hepatocellular carcinoma (HepG2) cells were used to observe the graphene oxide conjugated PEG (GOP), PCA and CA drugs, and the GOP-PCA/CA and GOP-PCA/CA-FA nanocomposites toxicity level. The HDFa dermal fibroblast cells were grown in a fibroblast basal medium with a Fibroblast Growth Kit–Serum-Free (ATCC^®^ PCS-201-040). Meanwhile, the HepG2 cells were grown in Eagle’s Minimum Essential Medium with 1% penicillin antibiotics containing 10,000 units/mL and 10,000 µg/mL of streptomycin (Nacalai Tesque, Kyoto, Japan). All the cells were well maintained and incubated at 95% humidity, 5% CO_2_ and 37.5 °C. The cells were harvested using 0.25% trypsin/1 mM-EDTA (Nacalai Tesque, Kyoto, Japan). In 96-well culture plates, 1.0 × 10^4^ cells/well were seeded for 24 h and treated with nanocarrier, nanocomposites and drugs for 72 h. After 72 h of treatment, 10 µL of MTT solutions (5 mg/mL in PBS) were added into each well and further incubated for 3 h and the supernatant were discarded. Then, 100 μL of dimethyl sulfoxide was added per well and incubated in the dark for 30 min until purple formazan salt was observed. The intensity of the purple formazan solution was measured at a wavelength of 570 nm using a microplate reader (Biotek LE800, Winooski, VT, USA). The IC50 value was plotted by x- against the y-axis and converted the x-axis values (concentration) to their log values, followed by nonlinear regression (curve fit) under the xy analysis to obtain a straight line equation fit, y = ax + b, from which the regression line and then inhibition IC50 was calculated.

% Cell viability = (Mean OD of individual test group)/(Mean OD of controlled group) × 100(3)

### 3.10. Lactate Dehydrogenase Assay for Plasma Membrane Integrity Analysis

The HepG2 cells were grown on a 96-well plate with a seeding density of 1 × 10^4^ cells/well overnight and treated with graphene oxide conjugated PEG (GOP), PCA and CA drugs, and GOP-PCA/CA and GOP-PCA/CA-FA nanocomposites according to different concentrations from 1.56–100 µg/mL and incubated for another 72 h. For the positive control, 10 µL of lysis buffer solution was added to untreated (control) cells prior to 45 min before taking the absorbance reading. From the treated cells, 50 µL of supernatant solutions were pipetted out into a new 96-well plate. This was followed by 50 µL of detection reagents being added and incubated for 30 min in the dark at room temperature. Finally, the 50 µL stop solution was added and the absorbance value for the lactate dehydrogenase was recorded at 490 nm on a microplate reader. The release percentage of LDH expression was calculated using the following calculation:

LDH release intensity ratio = Experimental LDH release (OD490)/Maximum LDH release (OD490)(4)

where Experimental LDH release is from cells treated with nanocomposites and Maximum LDH release is the positive control for LDH of the cells.

### 3.11. Morphological Observation of Nanocomposite Cellular Uptake

The HepG2 cells were cultured overnight with approximately 1.0 × 10^5^ cells/mL, and were treated with 40 µg/mL of drugs and nanocomposites for an incubation period of 72 h. Cells incubated with Eagle’s Minimum Essential medium were used as the negative control. The cell pellets were centrifuged and fixed with 2.5% glutaraldehyde in 0.1 M of sodium cacodylate buffer for 2 h at 4 °C, then washed with 0.1 M of sodium cacodylate buffer and post-fixed in 1% aqueous osmium tetroxide for 2 h at 4 °C. The samples were diced into 1 mm cubes and dehydrated in a series of acetone range from 30% to 100%. The samples were infiltrated with a 1:1 mixture of acetone and resin for 2 h and subsequently with 100% resin infiltrated overnight. The samples were transferred into embedding BEEM capsules and polymerized at 60 °C for 48 h. Finally, an ultrathin section of the resin-embedded tissue samples were obtained by using the ultra-microtome and transferred onto 400 mesh copper grids. The samples were stained with uranyl acetate and lead citrate and observed under the (JEOL JEM-2100F, Tokyo, Japan) transmission electron microscope.

### 3.12. In Vitro Localization of Nanocomposite by Fluorescent Microscopy Analysis

The cells were seeded into six-well plates at a density of 5.0 × 10^5^ cells/well. Once confluent, cells were treated with 200 µL of GOP-PCA/CA-FA and FITC-GOP-PCA/CA-FA and incubated for different time periods (0, 24, 72 h). The cells were washed with 1×PBS, followed by fixation using 4% formaldehyde and incubated for 3–5 min in an incubator. The fixatives were discarded and washed with 1×PBS. The cell nucleus was stained with DAPI solution (0.5 µg/mL). The cells were then further incubated for 5 min at room temperature. Finally, the DAPI stain was discarded and washed with 1×PBS. Fresh culture medium was added. The localization and accumulation of FITC-GOP-PCA/CA-FA was observed under an IX3P2F/Olympus fluorescence inverted microscope (Olympus, Hamburg, Germany).

### 3.13. Apoptosis Cell Death Analysis

The cells were cultured until the cell density of 1 × 10^6^ cells/mL was obtained. Then, they were treated with 40 µg/mL of graphene oxide conjugated PEG (GOP), PCA and CA drugs, and GOP-PCA/CA and GOP-PCA/CA-FA nanocomposites. Untreated cells were used as the negative control. After 72 h of treatment, cells were harvested by trypsinization, and centrifuged to collect cell pellets. Cell culture counted for 1 × 10^5^ cells/mL in 100 µL was counted and transferred into 5 mL flow tubes. Cell solutions were mixed with 5 µL of Annexin-V/FITC stain and 5 µL of PI stain. The flow tubes were gently vortexed and incubated for 15 min in the dark at room temperature. Finally, 400 µL of 1× binding buffer was added into individual flow tubes and the samples were analyzed in flow cytometry (BD FACS Canto II). Each quadrant was pooled into the sub-populations of healthy cells, early apoptotic cells, late apoptotic cells and necrotic cells.

### 3.14. Cell Cycle Arrest Analysis Using Propidium Iodide

The cell cycle arrest of HepG2 cells were determined by treating the cells with 40 µg/mL of pristine drugs and nanocomposites for 72 h. Untreated cells were used as the negative control. After 72 h of treatment cells were harvested using a scrapper and centrifuged to collect cell pellets. Cell culture counted for 1 × 10^5^ cells/mL in 100 µL was counted and transferred into 5 mL flow tubes. Cell solutions were resuspended with 250 µL of solution A and 250 µL of solution B from the CycleTEST TM PLUS DNA Reagent Kit. Finally, 200 µL of PI staining solution were added and incubated on ice for 10 min. The samples were analyzed in flow cytometry (BD FACS Canto II). The flow cytometry was programmed to read 10,000 events for each set of experiments. The cell cycle arrest results which presented in the sub G0/G1, G0/G1, S and G2/M phases were analyzed using Modfit software Version 3.2.

### 3.15. Mitochondrial Membrane Potential Analysis

Mitochondrial membrane potential analysis in the HepG2 cells were determined using JC-1 fluorescence staining assay. The cells were cultured until the cell density of 1 × 10^6^ cells/mL obtained. Then, they were treated with 40 µg/mL of the GOP nanocarrier, PCA and CA drugs, and GOP-PCA/CA and GOP-PCA/CA-FA nanocomposites. Untreated cells were used as the negative control and 10 µM of CCCP was treated as the positive control. After 72 h of treatment, cells were harvested by using a scrapper and centrifuged to collect cell pellets. The cell solutions for 5 × 10^5^ were counted and transferred to flow tubes and resuspended with 100 µL/mL of JC-1 stain. The flow tubes were incubated in 5% CO_2_ incubator for another 15 min. After incubation the cells were centrifuged at 400× *g* for 5 min and resuspended in 1 mL of JC-1 assay buffer and analyzed in flow cytometry (BD FACS Canto II).

### 3.16. Measurement of Cellular Reactive Oxygen Species

The HepG2 cells were seeded in a 96-well plate until the cells reached a density of 1 × 10^4^ cell/well. Then, they were treated with 40 µg/mL of drugs and nanocomposites for 72 h. Untreated cells were used as a negative control whereas the positive control was incubated with 200 mM H_2_O_2_ for 1 h before adding the DCFH-DA probe. After the treatment, the culture medium was replaced with 100 µL of 20 µM oxidant-sensitive dye DCFH-DA and incubated for 1 h at 37 °C. The cells were washed with a D-Hanks buffer solution and 100 µL of D-Hanks buffer solution was added into each well and the fluorescence intensity was recorded by a microplate reader at the excitation (435 nm) and emission (585 nm) wavelengths. The ROS fluorescence intensity ratio was calculated using the formula:

ROS intensity ratio = (F test-F blank)/(F control-F blank)(5)

where F test stands for fluorescence intensity of the treated cell or positive control, F control is fluorescence intensity of the untreated cell and F blank is fluorescence intensity of empty wells without cells.

### 3.17. Statistical Analysis

The regression analysis was plotted to calculate the dose–response relation where the mean ± SD for triplicates the IC50 values for MTT assays. All the data obtained from each assay were expressed as mean ± standard deviation (SD) from three independent experiments (*n* = 3 for each experiment). A normality test was performed using the Shapiro–Wilks test and Levene’s test was used for the homogeneity test of variance. Statistical analysis was performed using analysis of variance (ANOVA) followed by Games–Howell post hoc tests to observe the significant difference using SPSS program Version 22. Two variable data were analyzed using an unpaired *t*-test. A value of *p* ˂ 0.05 was considered to be statistically significant.

## 4. Conclusions

In this research, we have investigated the characterization—including in vitro release and encapsulation efficacy—of the dual drug loading of nanocomposites with passive and active targeting. We also concluded that dual drug loading nanocomposites drug delivery demonstrated an improvement in cell cytotoxicity properties. Cytotoxicity studies and lactate dehydrogenase assay proved the dose-dependent toxicity effect in both GOP-PCA/CA (passive targeting) and GOP-PCA/CA-FA (active targeting). The cellular drug uptake, localization and accumulation of the nanocomposites has also been observed. The nanocomposites force the HepG2 cells to undergo apoptosis at the late apoptosis phase. The HepG2 cells were arrested at the G2/M phase and depolarized the mitochondrial membrane potential while controlling the redox status. The formulation of GOP-PCA/CA-FA shows that the folic acid-coated dual drug loaded nanocomposite resulted in a significant reduction in cell cytotoxicity due to the combination drug loading and a controlled sustained release of PCA and CA compared to GOP-PCA/CA nanocomposite and pristine drugs.

## Figures and Tables

**Table 1:** Zeta potential were measured using a Zetasizer. FBS: fetal bovine serum.

Nanocomposites	ζ-Potential (mV)		
	Deionized Water	Eagle’s Minimum Essential Medium	Eagle’s Minimum Essential Medium + 10% FBS
GOP	−8.99 ± 2.021	−14.15 ± 1.224	−6.42 ± 1.302
GOP-PCA/CA	−17.10 ± 1.072	−22.19 ± 1.151	−15.44 ± 1.932
GOP-PCA/CA-FA	−22.72 ± 2.311	−29.28 ± 1.618	−19.76 ± 2.041

**Table 2:** The drug loading and encapsulation efficiency of nanocomposites.

Nanocomposites	Loading Content (%)		Encapsulation Efficiency (%)	
	PCA	CA	PCA	CA
GOP-PCA/CA	23.82	19.55	75.23	72.11
GOP-PCA/CA-FA	24.47	23.33	79.16	75.15

**Table 3:** The half-maximal inhibitory concentration (IC50) value of drugs and nanocomposites on HDFa dermal fibroblast cell and HepG2 cell lines.

Nanocomposites IC_50_ (μg/mL)	HDFa Dermal Fibroblast Cell	HepG2 Cell
Graphene oxide-PEG (GOP)	N.C	N.C
Protocatechuic acid (PCA)	N.C	40.78 ± 1.92
Chlorogenic acid (CA)	N.C	43.61 ± 1.74
GOP-PCA/CA	N.C	34.73 ± 1.04
GOP-PCA/CA-FA	N.C	26.79 ± 1.63

Abbreviation: N.C, No cytotoxicity.

## Data Availability

Not applicable.
